# Antioxidant, Anti-Tyrosinase, and Anti-Skin Pathogenic Bacterial Activities and Phytochemical Compositions of Corn Silk Extracts, and Stability of Corn Silk Facial Cream Product

**DOI:** 10.3390/antibiotics12091443

**Published:** 2023-09-13

**Authors:** Raenu Yucharoen, Pawalee Srisuksomwong, Jakaphun Julsrigival, Lapatrada Mungmai, Thida Kaewkod, Yingmanee Tragoolpua

**Affiliations:** 1 Division of Biology and Biotechnology, Faculty of Science and Technology, Nakhon Sawan Rajabhat University, Nakhon Sawan 60000, Thailand; raenu.y@nsru.ac.th; 2 Division of Science and Mathematics, Faculty of Science and Technology, Phuket Rajabhat University, Phuket 83000, Thailand; pawalee.s@pkru.ac.th; 3 Department of Pharmaceutical Sciences, Faculty of Pharmacy, Chiang Mai University, Chiang Mai 50200, Thailand; jakaphun.jul@cmu.ac.th; 4 Division of Cosmetic Science, School of Pharmaceutical Sciences, University of Phayao, Phayao 56000, Thailand; lapatrada.mu@up.ac.th; 5 Department of Biology, Faculty of Science, Chiang Mai University, Chiang Mai 50200, Thailand; thida.kaewkod@cmu.ac.th

**Keywords:** antibacterial, antioxidant, corn silk, cosmetic, formulation, tyrosinase

## Abstract

*Zea mays* L. Poaceae stigma (corn silk, CS) is a byproduct of agricultural waste and is used as a traditional herb in many countries. CS is rich in chemical compounds known to benefit human health and is also a remedy for infectious diseases and has anti-proliferative effects on human cancer cell lines. In the present study, CS extract has been evaluated for its antioxidant, antibacterial, and anti-tyrosinase activities and its phytochemical composition. The higher total phenolic and flavonoid contents were found in the ethanolic extract of corn silk (CSA), at 28.27 ± 0.86 mg gallic acid equivalent/g extract and 4.71 ± 0.79 mg quercetin equivalent/g extract, respectively. Moreover, the antioxidant content of CSA was found at 5.22 ± 0.87 and 13.20 ± 0.42 mg gallic acid equivalent/g extract using DPPH and reducing power assays. Furthermore, the ethanolic extract of corn silk showed tyrosinase inhibition with an IC_50_ value of 12.45 µg/mL. The bacterial growth inhibition of CSA was tested using agar disc diffusion and broth dilution assays against *Cutibacterium acnes* and *Staphylococcus epidermidis*. It was found that CSA inhibited *C. acnes* and *S. epidermidis* with an inhibition zone of 11.7 ± 1.2 and 9.3 ± 0.6 mm, respectively. Moreover, the CSA showed MIC/MBC of 15.625 mg/mL against *C. acnes*. The following phytochemical compounds were detected in CSA: cardiac glycosides; *n*-hexadecanoic acid; hexadecanoic acid, ethyl ester; oleic acid; and 9,12-octadecadienoic acid, ethyl ester. After the corn silk cream product was formulated, the product demonstrated stability without phase separation. This research is beneficial for promoting effective ways to use agricultural waste while utilizing the antioxidant, anti-tyrosinase, and antibacterial activities of corn silk. Moreover, the use of technology and innovation to obtain high-value CS extract will benefit the development of commercial cosmetic products by providing safe, natural, and quality ingredients to the consumer.

## 1. Introduction

The cause of major health problems in both humans and animals is air pollution, which is created from open burning, forest fires, and industrial emissions generating toxic substances. Furthermore, the open burning of agricultural residues to eliminate them after a harvest is a big source of aerosol emissions [1,2]. This burning is one way to manage rice, sugarcane, and corn residues; however, a large area of land engulfed by smoke greatly jeopardizes quality of life, as well as impacting climate change. Corn silk (*Zea mays* L., Poaceae family) refers to the stigma of the female flower of maize, which is discarded during the processing of corn products and considered agricultural waste. It contains many bioactive compounds such as protein, fiber, carbohydrates, minerals, vitamin A, and vitamin B2, which is full of polyphenol compounds with intense free radical scavenging activity [3]. It has been consumed as a traditional herbal medicine in China, Turkey, the United States, France, and some other countries.

In traditional medicine, corn silk is used to treat cystitis, edema, diabetes mellitus, gallstone disease, urinary tract infections, and prostatitis [4]. In addition, it has been found to contain phenolic acids, flavonoids, ascorbic acid, tannins, and cardiac glycosides [5]. Corn silk contains many bioactive compounds and antioxidant properties that benefit human health, and it can be used as a food ingredient [6]. The major compounds in corn silk, such as phenolic and flavonoid compounds and flavone glycosides, vary depending on the habitat of the plants and the extraction process [7,8]. The results obtained in this investigation revealed that the total antioxidant activity may be attributed to the presence of flavonoid and phenolic constituents in corn silk at the silking stage.

Moreover, corn silk extract also reduces UVB-induced skin damage [9] and acts as a pigment-reducing agent that regulates skin pigmentation by lowering tyrosinase expression [10]. Hyperpigmentation is a common skin condition and characterized by the darkening of certain areas of the skin due to an overproduction or accumulation of melanin [11]. Melanin is the pigment that determines the color of skin, hair, and eyes. It is produced by tyrosinase enzymes that are found in melanocytes. The tyrosinase enzyme plays a key role in the conversion of tyrosine into a substance called dihydroxyphenylalanine (DOPA) and the oxidation of DOPA to dopaquinone [12]. Therefore, anti-tyrosinase or skin-lightening agents such as kojic acid, vitamin C, hydroquinone, and arbutin are widely used to inhibit tyrosinase activity and act as active substances in skincare products [13,14]. Many plant extracts have a potent inhibitory effect on the production of melanin such as *Allium cepa*, *Aloe vera*, *Curcuma longa*, *Morus alba*, *Cymbopogon citratus*, or *Rosa canina* [15]. 

In addition, silver nanoparticles synthesized by the combination of parsley, corn silk, and gum arabic extract exhibit potent anti-inflammatory activity and are active against *Escherichia coli* MTCC 443 and *Pseudomonas aeruginosa* MTCC 1034 [16]. Human-resistant strains of bacteria are emerging in all countries, and more so in developing countries. For example, *Staphylococcus epidermidis* is the most frequent causative agent involved with infections at peripheral or central intravenous catheters. Staphylococcal infections can become resistant to antibiotic drugs [17]. Resistance development is also based on prevention, which includes medical equipment sterilization and contact during surgery [18,19].

*Cutibacterium acnes* and *Staphylococcus epidermidis* are major pathogens which cause a wide variety of skin and soft-tissue infections and are capable of causing life-threatening, invasive diseases. Antibiotics are found to be effective against inflammatory acnes, but antibiotic treatment has led to antibiotic resistance of up to 40%. *C*. *acnes* is considered an obligate anaerobe and Gram-positive bacteria. It has proteins required for oxidative phosphorylation, which possess cytochrome D oxidase genes. Propionic acid was produced by *C*. *acnes* which was caused by comedones. In this acidic pH, *C*. *acnes* can grow in human epithelial tissues for a long duration [20,21]. 

During *P. acnes* infection, ROS (reactive oxygen species) are produced by NADPH oxidase in keratinocytes and produce many pro-inflammatory molecules *in vitro*. *Acne vulgaris* is a multifactorial disorder involving sebaceous hyperkeratinization that is mediated by cytoplasmic NADPH oxidase and colonized by *C*. *acnes*. All target cells were stimulated by *C. acnes*, which can produce lytic enzymes in its environment, triggering the follicular epithelium’s disruption and activating the immune system. *C*. *acnes* can survive in many different sites within the tissue. It may secrete many antigenic components that are involved in its inflammation. *C*. *acnes* can produce many virulence factors and lead to an inflammatory process in the skin [22].

Nowadays, the cosmetics industry is quickly developing in many countries and appears to be the fastest-growing market in Asia [23]. Skin care products with natural ingredients are of higher interest to customers and are increasing in demand. Herbal extracts are primarily used in skincare formulations due to properties such as depigmentation, antimicrobial activity, and antioxidant capacity, which can be beneficial for the attenuation and prevention of various skin conditions. Furthermore, the extract might contain potentially harmful substances such as coumarins and furocoumarins from their phototoxic properties [24,25,26]. This study investigates the efficacy of corn silk on the inhibition of pathogenic bacteria, as well as the anti-tyrosinase activity, total phenolic content, total flavonoid compounds, antioxidant activities, and chemical composition of corn silk extracts. Additionally, the formulation and stability of cosmetic products containing corn silk extract are investigated.

## 2. Results

### 2.1. Corn Silk (CS) Extract Preparation

The crude extracts of corn silk were prepared via extraction using 95% ethanol and ethyl acetate. Among the different solvent extractions, CS extracted using ethanol gave a yield percentage higher than CS extracted using ethyl acetate, with the values of 18.9 and 15.5%, respectively.

### 2.2. Total Phenolic and Flavonoid Contents of CS Extract

The ethanolic and ethyl acetate extracts of CS showed a phenolic content of 28.27 ± 0.86 and 12.81 ± 0.17 mg gallic acid equivalent/g extract. Moreover, the total flavonoid content of ethanolic and ethyl acetate extracts of CS were 4.71 ± 0.79 and 2.23 ± 0.57 mg quercetin equivalent/g extract, respectively (Table 1).

### 2.3. Phytochemical Profiling

The phytochemical composition of CS extract was analyzed, and cardiac glycosides were detected in the ethanolic and ethyl acetate extracts of corn silk, while alkaloid, saponin glycoside, cyanogenic glycoside, antraquinone glycoside, flavonol, flavone, tannin, and coumarin were not found. These bioactive compounds have been known to have medicinal properties as well as exhibit physiological activities that are useful to humans.

### 2.4. GC/MS Analysis

**Figure 1:**
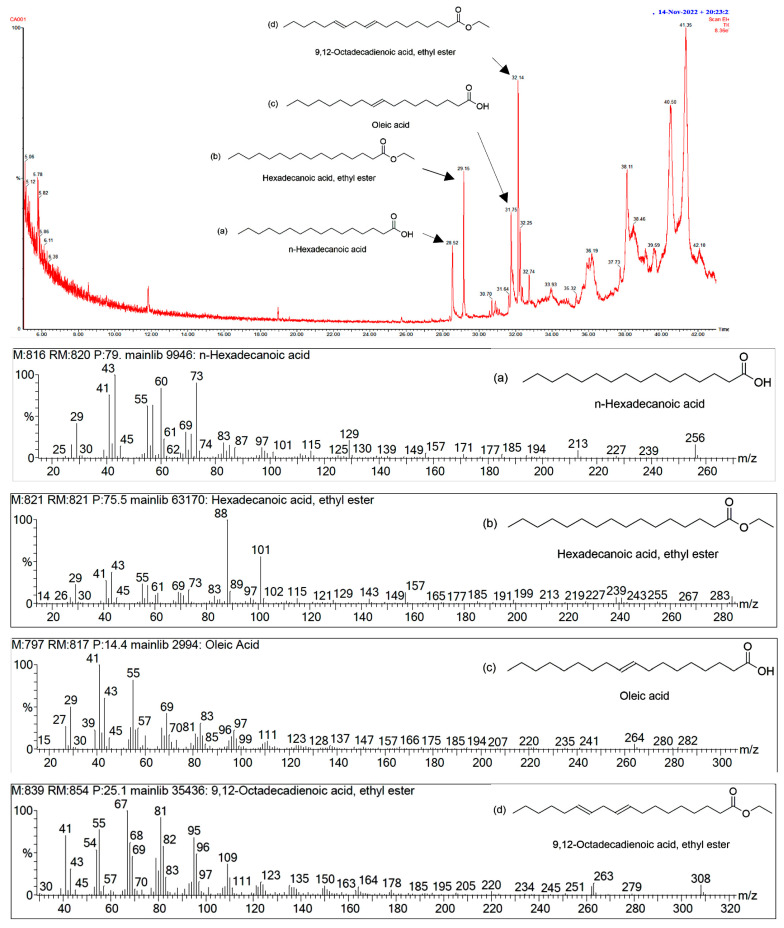
GC/MS chromatogram and spectra of volatile organic compounds released from the ethanolic extract of CS. The peak at 28.52 min represents *n*-hexadecanoic acid (**a**), and the peak at 29.15 min represents hexadecanoic acid, ethyl ester (**b**). The peak at 31.75 min represents oleic acid (**c**), and the peak at 32.14 min represents 9,12-octadecadienoic acid, ethyl ester (**d**).

The main volatile compounds were identified using GC/MS. A GC/MS profile revealed the major compounds with the percentages of peak area at retention times of 28.52 min, 29.15 min, 31.75 min, and 32.14 min, which were tentatively identified as *n*-hexadecanoic acid (1.36%); hexadecanoic acid, ethyl ester (2.13%); oleic acid (1.77%); and 9,12-octadecadienoic acid, ethyl ester (2.62%), respectively, in an ethanolic extract of CS (Figure 1).

### 2.5. Antioxidant Activity Using DPPH and Reducing Power Assays of CS Extract

Antioxidant activity was determined using DPPH radical scavenging and reducing power assays in this study. The ethanolic extract of CS confirmed an antioxidant activity of 5.22 ± 0.87 mg gallic acid equivalent/g extract. Moreover, the ethyl acetate extract of CS showed an antioxidant activity of 5.19 ± 0.37 mg gallic acid equivalent/g extract via DPPH assay. In addition, the ethanolic extract of CS exhibited the significantly higher reducing power of 13.20 ± 0.42 mg gallic acid equivalent/g extract (Table 2). The ethanolic extract of CS showed antioxidant activity, total phenolic content, and total flavonoid content significantly greater than the ethyl acetate of CS extract with *p*-value < 0.05 (Table 1 and Table 2). The results obtained in this investigation revealed that antioxidant activities including DPPH and reducing power assays may be attributed to the presence of phenolic and flavonoid constituents.

### 2.6. Anti-Tyrosinase Activity of Ethanolic Extract of Corn Silk

In this study, the ethanolic extract of corn silk (CSA) revealed the higher antioxidant activity than ethyl acetate extract. Therefore, the CSA was selected to study tyrosinase inhibition. Corn silk and kojic acid exhibited IC_50_ values of 12.45 and 4.85 µg/mL, respectively. 

### 2.7. Antibacterial Activities of Ethanolic Extract of Corn Silk

The ethanolic extract of CS (CSA) was chosen for further investigation since it exhibited stronger antioxidant activity. The efficacy and optimal concentration of the CSA for the inhibition of pathogenic bacteria was investigated. The growth inhibition of the CSA was tested using agar disc diffusion and broth dilution assays against *C*. *acnes* and *S*. *epidermidis*. It was found that the CSA showed higher antibacterial activity on *C. acnes* with an inhibition zone of 11.7 ± 1.2 mm (Table 3 and Figure S1), and MIC and MBC of 15.625 mg/mL (Table 4). In addition, gentamycin antibiotic showed MIC and MBC values of 0.0625 and 0.0156 mg/mL against *C. acnes* and *S. epidermidis*, respectively. In this study, the ethanolic extract of corn silk showed potential antioxidant activities along with anti-tyrosinase activity. Thus, these antibacterial activities should be fostered and applied to cosmetic skincare. 

### 2.8. Stability Test of Nourishing Corn Silk Cream

The cream formulation containing 1% CSA extract was prepared in this study, and its physical characteristics were stable under various conditions tested at room temperature, 4 °C, and 45 °C for 3 months and 6 cycles of the heating/cooling process (45 °C, 48 h and 4 °C, 48 h for 1 cycle). The physical characteristics and color remained the same. However, the viscosity changed slightly. The cream texture was stable without phase separation. The stability of the CSA cream after heating/cooling cycle remained within acceptable limits (Table 5).

## 3. Discussion

In this study, sweet corn silk was chosen at its mature state, as it has been previously reported to exhibit variability in phytochemicals such as phenolics, flavonoids, and anthocyanins [6]. CS was extracted with 95% ethanol and ethyl acetate, although a previous study showed that corn silk phytochemical compounds were extracted more often in high-polarity solvents [5]. However, these two solvents, 95% ethanol and ethyl acetate, have not been used to extract corn silk. Thus, these solvents have been chosen to determine the most effective solvent for the extraction which obtains the highest yield and most biological activities. 

The antioxidant activities of the CS ethanolic extract were also evaluated from previous studies using DPPH radical scavenging activity [15,27,28]. Ionizing radiation can cause harmful effects in the biological system resulting in the production of free radicals and ROS, such as hydroxyl radical and superoxide anion [6,27]. Hence, the antioxidant activity of four *Z. mays* varieties, including var. *intendata*, var. *indurata*, var. *everta*, and var. *saccharata*, was evaluated using DPPH, superoxide scavenging, iron chelating capacity, and ferric-reducing antioxidant power assay. The DPPH radical-scavenging assay indicated the electron donation ability of extracts and was able to be measured by DPPH^+^ solution bleaching. The ethanolic extract of four *Z. mays* varieties at 500, 1000, and 2000 µg/mL exhibited lower DPPH radical scavenging activity by 10–23%. Meanwhile, the ethanolic extract of *Z. mays* var. *intendata* showed strong antioxidant activity through iron chelating capacity. However, the ethanolic extract of Egyptian CS at 400 µg/mL could inhibit DPPH activity by 84% [28,29]. 

In China, the flavonoid 4′,5,7-trihydroxy-3′,5′-dimethoxyflavone 7-O-[β-D-api-furanosyl (1→2)]-β-D-glucopyranoside was isolated from the bract of *Z. mays* L. [30]. The obtained data from the antioxidant experiments in this study are in concordance with Liu et al. [16], who presented a relationship between compounds and antioxidant activity. The phenolic and flavonoid compounds were considered to be secondary metabolites and are known to exhibit health-promoting activities as antioxidant agents [30,31,32]. This com-pound may be responsible for the possible antioxidant activity from many natural sources, especially plants. Phenolic compounds are able to scavenge ROS, which causes various diseases such as cancer, hypertension, and cognitive disfunction. In addition, CS contains the ascorbic acid, tannins, and cardiac glycosides that could contribute to the antioxidant capacity of CS [5].

Moreover, CS from the stigmata of female maize flowers, which is agricultural waste from corn cultivation, [2,33] has been used as traditional medicine and for preventing diseases such as kidney stones, edema, cystitis, diuretic prostate disorder, urinary infection, and obesity. It induces diuresis and kaliuresis effects in Wistar rats [34,35], has anti-depressant and antioxidant activities against γ-radiation, and protects from oxidative stress in mice [36,37]. It has the potential to reduce hyperglycemia and nephrotoxicity in mice [38,39] and also carries anti-hyperlipidemic, anti-diabetic, and anti-inflammatory effects [4].

Another publication shows that the tyrosinase inhibition of CS extract revealed the highest IC_50_ value of 3082.28 ± 347.98 µg/mL in waxy corn silk [40]. Thus, corn silk demonstrated the efficacy of inhibiting the tyrosinase enzyme. It catalyzed the production of melanin in hair bulbs, the eyes of animals, and skin. Therefore, the CSA showed inhibitory effects on the tyrosinase enzyme as a skin whitening agent. The CSA has also demonstrated tyrosinase-inhibiting activity, which could be used for cosmetic applications [41].

Many researchers have reported tyrosinase inhibitors derived from medicinal plants used for skin products. Kishore et al. [42] and Söhretoqlu et al. [43] referenced flavonoids and quercetin, which are associated with tyrosinase-inhibitory effects. Tyrosinase is a copper-containing monooxygenase enzyme, which catalyzes melanin biosynthesis, including the ortho-hydroxylation of amino acid, tyrosinase, to DOPA, and the oxidation of DOPA to ortho-quinone [44,45]. 

In this study, the CSA demonstrates an anti-bacterial effect against Gram-positive bacteria. Although the MIC/MBC values of corn silk extracts are not so strong, other additional biological activities such as antioxidant and anti-tyrosinase activities were also found in the corn silk extracts. Previous publications have reported the antibacterial effect of CS, and the activity of CS possibly correlated with the presence of phenolics and flavonoids [46,47]. Cardiac glycosides consist of a steroid molecule attached to a sugar (glycoside) and an R group that is also found in plants [48]. The study by Ali et al. (2019) demonstrated that cardiac glycosides, flavonoid glycosides, terpenoids, steroidal compounds, alkaloids, and saponins isolated from *Tamarix aphylla* leaves showed antifungal, antibacterial, anti-inflammation, antioxidant, antidiabetic, hypolipidemic, and wound-healing properties [49]. However, the cytotoxicity of corn silk extracts will be investigated in human epithelial cells in the future.

Furthermore, the bioactive compounds in corn silk, including *n*-hexadecanoic acid, hexadecanoic acid, ethyl ester, oleic acid, and 9,12-octadecadienoic acid, ethyl ester were detected by GC/MS. These compounds were similar to the study by Abirami et al. (2021), and also found in the ethanolic extract [50]. The *n*-hexadecanoic acid is mostly present in plants that exhibit biological properties such as anti-inflammatory, antioxidant, and antibacterial activities [51,52]. Hexadecanoic acid, ethyl ester showed biological properties such as antioxidant, antibacterial, and antiandrogenic activities [53,54]. Oleic acid has demonstrated the ability to prevent cancer and act as an antiandrogenic [55]. Additionally, 9,12-octadecadienoic acid, ethyl ester also exhibited anticancer and anti-inflammatory properties [56]. Thus, volatile compounds found in CS possess various biological activities.

In addition, corn silk is rich in phenolic compounds and consists of proteins, vitamins, carbohydrates, calcium, potassium, magnesium, volatile oils, sitosterol, stigmasterol, alkaloids, and saponins [15]. The phytochemical composition of CS extracts is mainly due to the flavonoid content: 6,4′-dihydroxy-3′-methoxyflavone-7-O-glycosides ax-5″-methane-3′-methoxymaysin, ax-4″-OH-3′-methoxymaysin, 7,4′-dihydroxy-3′-methoxyflavone-2″-O-α-L-rhamno-syl-6-C-fucoside 3′-methoxymaysin, 2″-O-α-L-rhamnosyl-6-C-fuco-syl-3′-methoxyluteolin, 2″-O-α-L-rhamnosyl-6-C-quinovosylluteolin, 2″-O-α-L-rhamno-syl-6-C-fucosylluteolin, and 2″-O-α-L-rhamnosyl-6-C-3″-deoxyglucosyl-3′-methoxyluteolin have been identified [55,56]. Meanwhile, the flavone glycoside content includes isoorientin-2-2″-O-α-L-rhamnoside and 3′-methoxymaysin [16]. Moreover, the volatile dichloromethane compound of Egyptian CS, including terpenoids, cis-α-terpeneol, citronellol, 6,11-oxidoacor-4-ene, trans-pinocamphone, eugenol, neo-iso-3-thujanol and cis-sabinene hydrate, was identified using gas chromatography–mass spectrometry (GC-MS) [4,28].

Other studies reported that 100 ppm of CS extract decreased melanin production in Melan-A cells by 37.2% without cytotoxicity. The application of CS extract on female faces with hyperpigmentation significantly reduced skin pigmentation without abnormal reactions after the application of 0.75 and 1.5% solution of CS extract twice a day for 8 weeks [12]. This research indicates that the uses of agricultural waste, including corn silk, have great potential to produce value-added products and reduce environmental problems. Moreover, it is an attractive material for the pharmaceutical and food industries. This research is also beneficial in promoting effective use of agricultural waste including corn silk, through technology and innovation, which is beneficial to the local community and increases the standards of cosmetic products.

## 4. Materials and Methods

### 4.1. Sample Collection and Corn Silk (CS) Extract Preparation

Sweet corn silk was collected at 20 days after anthesis (immature stage) from Khok Phra District, Nakhon Sawan Province, Thailand, in January 2020. The samples of fresh corn silk (CS) were cleaned, dried under room temperature, and extracted with ethanol or ethyl acetate via maceration for 7 days. The ratio of sample to solvent (*w*/*v*) for extraction was 1:20. Extracts were filtered through Whatman No. 1 filter paper. The solvent was evaporated, and the filtrate was concentrated using a rotary evaporator. Next, the extracts were lyophilized to obtain a dried powder and were dissolved in 10% dimethyl sulfoxide (DMSO) to prepare the solution of the extract before use.

### 4.2. Determination of Total Phenolic Content

The total phenolic content was determined using the Folin–Ciocalteu method [57]. The reaction mixture was combined with 250 µL of 95% ethanol, 1.25 mL of deionized water, 250 µL of ethanolic or ethyl acetate extract of CS, and 125 µL of 50% Folin–Ciocalteu reagent (Merck, Darmstadt, Germany). After incubation for 5 min, 250 µL of 5% Na_2_CO_3_ was added to the mixture and incubated in the dark at room temperature for 1 h. The absorbance was measured at 725 nm. Gallic acid was used as a standard. The phenolic content was calculated and is expressed as mg gallic acid equivalent/g extract.

### 4.3. Determination of Total Flavonoid Content

The flavonoid compounds were determined using aluminum chloride colorimetric assay [57]. A total of 500 µL of ethanolic or ethyl acetate extract of CS were added to 100 µL of 10% aluminum chloride. Then, 1.5 mL of methanol, 100 µL of 1 M potassium acetate, and 2.8 mL of deionized water were mixed. The mixture was incubated at room temperature for 30 min. The absorbance was measured at 415 nm. Quercetin was used as a standard. The flavonoid content was calculated and is expressed as mg quercetin equivalent/g extract.

### 4.4. Phytochemical Profile of CS Extract

The ethanolic extract of corn silk was dissolved in methanol and subjected to a preliminary screening for phytochemical constituents using standard procedures. A quantitative test was performed to determine alkaloid, tannin, and glycoside content as described by Obadoni and Ochuko [58], with slight modifications.

### 4.5. GC/MS Analysis of CS Extract

The CS extract was dissolved in dichloromethane and dimethyl sulfoxide. The separation and identification of volatile compounds were conducted through GC/MS. GC/MS analysis was performed with a Clarus Model 690 gas chromatograph (PerkinElmer, Waltham, MA, USA) coupled to a model SQ8 mass-selective detector. The capillary column used was a PerkinElmer Elite-5MS (5% phenylmethyl polysiloxane with a 30 m × 250 μm ID × 0.25 μm film thickness) (PerkinElmer, Waltham, MA, USA). The injector temperature was 250 °C. The oven temperature was initially held at 60 °C for 5 min; then, it was increased at a ratio of 5 °C/min to a final temperature of 250 °C and held for 5 min. The carrier gas utilized was helium gas with a purity of 99.999%. Electron impact (EI) mass spectra were collected at 70 eV ionization voltages over the range of *m*/*z* 60–600. The electron multiplier voltage was 1250 V. Both ion source and quadruple temperatures were set at 200 °C.

The identification of volatile organic compounds was accomplished by comparing their mass spectra with those in the National Institute of Standards and Technology (NIST) Library Search Chromatogram version 2020. Additional identification was performed by assessing the matching percentage (match factor—M and reverse match factor—RM), which needed to be greater than or equal to 80% in comparison to the known compounds in the NIST library. 

### 4.6. Antioxidant Activity

#### 4.6.1. DPPH Radical Scavenging Activity Assay

2,2′diphenyl-l-picrylhydrazyl (DPPH) radical scavenging was measured using the method of Hou et al. [59] with some modifications. Various concentrations of ethanolic or ethyl acetate extract of CS were mixed with 1.5 mL of 0.1 mM DPPH solution (Sigma Aldrich Chemical, St. Louis, MO, USA). The reaction mixture was kept in the dark at room temperature for 20 min, and the absorbance was measured by spectrophotometer at 517 nm. Gallic acid was used as an antioxidant standard. A decrease in absorbance at 517 nm was determined. The percentage of free radical inhibition was determined using the following formula: [(A_control_ − A_sample_)/A_control_] × 100. Afterward, the concentration of the extract and gallic acid necessary to achieve 50% inhibition of the free radical was calculated. The antioxidant activity was quantified as milligrams of gallic acid equivalent per gram of the extract (mg GAE/g extract).

#### 4.6.2. Reducing Power Assay

The reducing power of ethanolic or ethyl acetate extract of CS was determined according to the modified method of Oyaizu [60]. A total of 120 μL of the sample was mixed with 290 μL of 0.2 M phosphate buffer (pH 6.6) and 290 μL of 1% *w/v* potassium ferricyanide. The mixture was incubated at 50 °C for 20 min. Next, 290 μL of 10% TCA was added and mixed with 1 mL of deionized water and 0.2 mL of 0.1% FeCl_3_. The absorbance was measured at 700 nm. The percentage of free radical inhibition was determined using the following formula: [(A_control_ − A_sample_)/A_control_] × 100. Afterward, the concentration of the extract and gallic acid necessary to achieve 50% inhibition of the free radical was calculated. The antioxidant activity was quantified as milligrams of gallic acid equivalent per gram of the extract (mg GAE/g extract).

### 4.7. Determination of Corn Silk’s Anti-Tyrosinase Activity 

The anti-tyrosinase activity was determined using the dopachrome micro-plate [61]. The ethanolic extract of CS was diluted with 50% (*w*/*w*) dimethyl sulfoxide. A total of 50 µL of the solution was combined with 150 µL of 0.02 M phosphate buffer (pH 6.8) and 50 µL of 313 Units/mL mushroom tyrosinase (Sigma Aldrich Chemical). Next, 50 µL of 0.32 mM 3,4-dihydroxy-L-phenylalanine (Sigma Aldrich Chemical) was used as a substrate and added to each well. The anti-tyrosinase activity was evaluated by measuring the absorbance at 492 nm using a microplate reader before incubation at 25 °C, and after incubation for 2 min. Kojic acid (Merck Millipore) was used as the standard inhibitor of the tyrosinase enzyme. The percentage of tyrosinase inhibition was calculated using this equation:

% Tyrosinase inhibition = {[(A − B) − (C − D)]/A − B} × 100(1)

where A is OD_492_ of control; B represents the blank control; C represents the sample reaction of L-DOPA with tyrosinase enzyme and CS extract in buffer; and D represents the blank sample of C.

### 4.8. Antibacterial Activity

The stronger antioxidant activity of the CS extract was chosen and investigated for growth inhibition against skin pathogenic bacteria using the agar disc diffusion method [62]. Skin pathogenic bacteria including *C. acnes* DMST 14,916 and *S. epidermidis* were used in this experiment. A single colony of *S. epidermidis* was cultivated in Muller Hinton (MH) broth and incubated at 37 °C for 18–24 h under aerobic conditions, whereas *C. acne* was cultivated in Brain Heart Infusion (BHI) Broth at 37 °C for 48–72 h under anaerobic conditions. The bacterial inoculum was adjusted to McFarland No. 0.5 (1.5 × 10^8^ CFU/mL) and swabbed on MH agar or BHI agar. A paper disc was soaked in 500 mg/mL of CSA and placed on the agar. Gentamycin and dimethyl sulfoxide (DMSO) were used as positive and negative controls, respectively. Experiments were performed in triplicate. After incubation, the antibacterial activity was indicated by the inhibition zone, which was measured after treatment [63].

### 4.9. Determination of the Minimum Inhibitory Concentration (MIC) and Minimum Bacterial Concentration (MBC)

The MIC and MBC of ethanolic and ethyl acetate extracts of CS were measured using the broth dilution method [63]. The CS extract was serial two-fold diluted in MH broth or BHI broth medium. The ethanolic or ethyl acetate extract of CS at the highest concentration (1000 mg/mL) was added to the first tube, then two-fold serial dilution was performed by transferring 0.5 mL of suspension to the subsequent tubes. Then, 0.5 mL of bacterial inoculum adjusted to McFarland No. 0.5 (1.5 × 10^8^ CFU/mL) was added into each concentration of the extracts. Gentamycin was used as the positive control. The treatment of *S. epidermidis* was then incubated at 37 °C for 18–24 h under aerobic conditions, except for *C. acnes*, which was incubated at 37 °C for 48–72 h under anaerobic conditions. The MIC is defined as the lowest concentration of extract that inhibits the growth of bacteria. For MBC determination, 0.01 mL of the extract from the MIC test tubes with no growth was streaked on agar plates. After incubation, the MBC is the lowest concentration to inhibit the growth of bacteria by 99.9%.

### 4.10. Formulation of Nourishing Corn Silk Cream Preparation

The conventional process of preparation of the cream formulation was separated into four parts: part A (water phase), including butylene glycol, carbomer, and deionized water; part B (oil phase), including glyceryl stearate, PEG-100 stearate, cetyl alcohol, and C12-15 alkyl benzo-ate; part C (additive), including HDI/trimethylol hexyllactone crosspolymer and silica. Moreover, the extract was dissolved in propylene glycol. In addition, caprylhydroxamic acid and 1,2-hexanediol were used as preservative reagents; Part D (neutralizer), including triethanolamine. The water phase of part A was heated at 70–75 °C, and the oil phase of part B was heated at 75–80 °C. Then, parts A and B were mixed using a homogenizer. After that, the mixture was cooled to 45–50 °C and parts C and D were added. The emulsion was continuously homogenized. Then, 1% CSA extract prepared in propylene glycol was added and mixed well until the mixture cooled down to room temperature.

### 4.11. Stability Studies of Corn Silk Cream Product

The corn silk cream product was subjected to centrifugation at 6000 rpm at 20 °C for 30 min to determine the phase separation of the product. Stability of the corn silk cream product was evaluated using the heating/cooling (H/C) process. The cream product was kept at room temperature, 4 °C, and 45 °C for 3 months, and 6 cycles of the heating/cooling process (45 °C, 48 h and 4 °C, 48 h for 1 cycle) were performed to determine the stability of the cream product. The changes in physiochemical appearance were then observed, including color, pH, and odor. The viscosity of the product was also determined using a viscometer at 10 rpm [62].

### 4.12. Statistical Analysis

Statistical analysis was performed using Microsoft Excel. All experiments were carried out in triplicate. Data values are expressed as mean ± standard deviation. The significant differences of the antioxidants were determined by an independent *t*-test group with *p*-value < 0.05.

## 5. Conclusions

In this study, corn silk extract showed tyrosinase inhibition, antioxidant activities, and antibacterial activities. The results revealed that skin bacteria including *C. acnes* and *S. epidermidis* were inhibited by the ethanolic extract of corn silk. In addition, the CSA exhibited higher antioxidant activities using DPPH and reducing power assays. The higher total phenolic and flavonoid contents were also found in the CSA. Moreover, cardiac glycosides, *n*-hexadecanoic acid, hexadecanoic acid, ethyl ester, oleic acid, and 9,12-octadecadienoic acid, ethyl ester were identified in the CSA. Cream product containing the CSA was proven to be stable at various storage conditions. Hence, the results indicate that the application of corn silk extract has great potential to be used as an added value to cosmetic products that could play a role in anti-aging and skin-whitening. However, active compounds from the CSA and the cytotoxicity of formulated cream product should be further investigated in the future.

## Figures and Tables

**Table 1:** Total phenolic and total flavonoid contents of ethanolic and ethyl acetate extracts of corn silk.

Biological Activity	Ethanolic Extract	Ethyl Acetate Extract
Total phenolic content (mg gallic acid equivalent/g extract)	28.27 ± 0.86 *	12.81 ± 0.17
Flavonoid content (mg quercetin equivalent/g extract)	4.71 ± 0.79 *	2.23 ± 0.57

ND = not detectable (mean ± standard deviation, *n* = 3). * Significant difference between ethanolic extract and ethyl acetate extract (*p*-value < 0.05).

**Table 2:** Antioxidant activity of ethanolic and ethyl acetate extracts of corn silk.

Antioxidant Activity	Ethanolic Extract	Ethyl Acetate Extract
DPPH radical scavenging (mg gallic acid equivalent/g extract)	5.22 ± 0.87	5.19 ± 0.37
Reducing power assay (mg gallic acid equivalent/g extract)	13.20 ± 0.42 *	1.09 ± 0.17

ND = not detectable (mean ± standard deviation, *n* = 3). * Significant difference between ethanolic extract and ethyl acetate extract (*p*-value < 0.05).

**Table 3:** Inhibitory effect of ethanolic extract of CS on skin bacteria using the agar disc diffusion method.

Treatment	Diameter of Inhibition Zone on Bacteria (mm)	
	*C.* *acnes*	*S.* *epidermidis*
Ethanolic extract of CS, 500 mg/mL	11.7 ± 1.2	9.30 ± 0.6
Gentamycin, 1 mg/mL	23.3 ± 0.6	23.7 ± 0.6

**Table 4:** Minimal inhibitory concentration (MIC) and minimal bacterial concentration (MBC) values of CSA against skin bacteria.

Treatment	*C. acnes*		*S. epidermidis*	
	MIC (mg/mL)	MBC (mg/mL)	MIC (mg/mL)	MBC (mg/mL)
CSA	15.625	15.625	125	125
Gentamycin	0.0625	0.0625	0.0156	0.0156

**Table 5:** The stability testing results of CSA cream after three months and after heating/cooling for six cycles.

Conditions	Color	pH	Viscosity (Pa·s/cP)	Centrifugation Test	Phase Separation	Homogeneity
RT	Pale yellow	7.60	4395.5	Stable	No	Good
4 °C	Pale yellow	7.41	4551.0	Stable	No	Good
45 °C	Pale yellow	7.13	6181.5	Stable	No	Good
H/C	Pale yellow	7.57	5500.0	Stable	No	Good

## Data Availability

Data are contained within the article.

## Supplementary Materials

The following supporting information can be downloaded at: https://www.mdpi.com/article/10.3390/antibiotics12091443/s1, Figure S1. Antibacterial activity of the ethanolic extract of CS by agar disc diffusion method against *C. acnes* and *S. epidermidis*. Ethanolic extract of CS, 500 mg/mL (1), gentamycin, 0.1 mg/mL (2) and DMSO (3).
