# Supplementary material for: Antioxidant, Anti-Tyrosinase, and Anti-Skin Pathogenic Bacterial Activities and Phytochemical Compositions of Corn Silk Extracts, and Stability of Corn Silk Facial Cream Product
Source: Antibiotics (Basel). 2023 Sep 13;12(9):1443. doi: 10.3390/antibiotics12091443 (PMC10525379; doi:10.3390/antibiotics12091443)
Supplement: Supplementary file 1 [file antibiotics-12-01443-s001.zip › antibiotics-2572107-supplementary.pdf]

Supplementary Materials

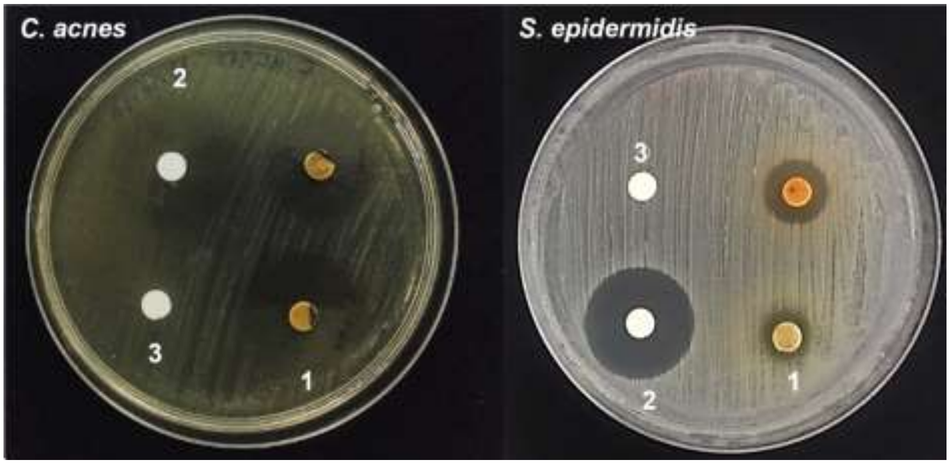

Figure S1. Antibacterial activity of the ethanolic extract of CS by agar disc diffusion method against *C. acnes* and *S. epidermidis*. Ethanolic extract of CS, 500 mg/mL (1), gentamycin, 0.1 mg/mL (2) and DMSO (3).
